# Special Issue—“Diseases of the Salivary Glands”

**DOI:** 10.3390/jcm9123886

**Published:** 2020-11-30

**Authors:** Margherita Sisto

**Affiliations:** Department of Basic Medical Sciences, Neurosciences and Sense Organs, Section of Human Anatomy and Histology, Laboratory of Cell Biology, University of Bari Medical School, 70124 Bari, Italy; margherita.sisto@uniba.it

## Editorial

Salivary glands (SGs) are of the utmost importance for maintaining the health of the oral cavity and carrying out physiological functions such as mastication, protection of teeth, perception of food taste, and speech. SGs may be affected by a number of diseases—local and systemic—and the prevalence of SG diseases depends on various etiological factors. The glands can be blocked by small stones that form in the gland ducts, which may cause painful swelling. The glands may become infected by viral, bacterial, or (rarely) fungal agents, or they may be the targets of autoimmune attacks that affect their functions. Management of salivary disorders encompasses a broad array of diseases—both benign and malignant. To better demonstrate the evolution of this field, the present Special Issue in the *Journal of Clinical Medicine* aims to cover recent and novel advancements as well as future trends in the field of research evaluating diagnostic and therapeutic treatment of SG diseases, reflecting the diverse nature of SG anatomy, physiology, and dysfunction in various states of disease. 

Several interesting findings are derived from this collective body of work. Firstly, in alignment with the most advanced diagnostic techniques for the identification of pathologies affecting the SGs, Tanaka et al. [1] reported the effective application of MR sialography and dynamic MR sialography to visualize the sublingual gland ducts, demonstrating, successfully, that MR sialography can be more useful in the diagnosis of patients with lesions of sublingual gland ducts. Van Ginkel and colleagues [2] enriched the Special Issue by contributing a descriptive review discussing imaging techniques that are used in the identification and characterization of primary Sjögren’s syndrome (pSS) with a focus on the SGs. Primary Sjögren’s syndrome (pSS) is a systemic autoimmune disease characterized by dysfunction and lymphocytic infiltration of the salivary and lacrimal glands, with unknown etiology. The review emphasizes the contribution of innovative techniques to the diagnosis of pSS and the monitoring of the progression of the disease, underlining the contribution to diagnosis and staging of pSS-associated lymphomas. Several of the papers published in this Special Issue provide insights into the etiology of pSS, highlighting aspects that contribute to the pathophysiology of the disease and exploring treatment options that target different mediators of the pSS pathogenesis. An important experimental contribution is given by the development of a pSS experimental model represented by immortalized SG epithelial cells derived from labial salivary gland biopsies of pSS patients [3]; these cells represent a viable model for salivary research due to their passaging capacity and maintenance of acinar cell characteristics. The etiology of pSS remains poorly understood, but recent findings highlight the involvement of both immune system cells and various types of glandular cells that cooperate in the perpetuation of chronic inflammatory conditions that characterize pSS. Witas et al. [4] summarized the evidence for participation in disease pathogenesis by both various classes of immune cells and glandular epithelial cells in an interesting review collecting data from both preclinical mouse models and human patients. Relatedly, my research group [5] collected from the literature the most recent findings concerning the involvement of the transcription factor nuclear factor κ (kappa)-light-chain-enhancer of activated B cells (NF-κB) in the chronic inflammatory mechanisms implicated in the pathogenesis of pSS. To enrich this panorama of possible mechanisms underlying the pSS disease, Paris and colleagues [6] contributed a comprehensive review which addresses the clinical presentation, diagnosis, and complications of the disease, focusing on the fundamental role played by epithelial cells in autoimmune mechanisms and on the genetic, environmental, and hormonal features that represent risk factors for the disease. The important article by Nakamura et al. [7], which suggests and explains a viral etiology at the basis of pSS, fits well into this scenario. Nakamura delved into the role played by various viruses such as Epstein–Barr virus (EBV) or human T-cell leukemia virus type 1 (HTLV-1) that, regardless of the different cellular targets that they have, are involved in pathological immune modifications in pSS.

Finally, a part of the Special Issue is dedicated to the analysis of interesting case reports related to primary or secondary diseases of the SGs. The research group of Drs. Ingravallo and Capodiferro reported a clinical study conducted on a series of intraoral mucoepidermoid carcinomas showing exclusive intracystic growth [8] and a rare case of primary breast lymphoma occurring in a woman with long-standing pSS [9]. Morawska and colleagues performed a study on women with Hashimoto’s thyroiditis (HT) that evaluated the redox homeostasis of the SGs; data collected demonstrate that salivary secretion is impaired in patients with HT, and the release of oxidant molecules was found to be significantly higher in patients with HT than in healthy controls [10]. From the same research group, Maciejczyk et al. evaluated protein glycooxidation products, lipid oxidative damage, and nitrosative stress in chronic kidney disease (CKD) condition. Interestingly, their analysis indicates that children with CKD suffer from SG dysfunction that increases with CKD progression [11]. 

Given these diverse contributions, it is evident that research on SG diseases will continue to flourish. There are still many fundamental questions that remain unanswered, promising a great future for this field. As the Guest Editor, I would like to give special thanks to the reviewers for their timely and professional comments and to the members of the *JCM* Editorial Office for their robust support. Finally, I sincerely thank all the authors for their valuable contributions. I believe the readers of this Special Issue will find them very useful.
